# The occurrence of *Rodentolepis nana* and *Hymenolepis diminuta* in free-living rodents from Slovakia: The first molecularly confirmed records

**DOI:** 10.1016/j.crpvbd.2026.100404

**Published:** 2026-06-23

**Authors:** Daniela Antolová, Yaroslav Syrota, Júlia Halapy, Michal Stanko, Gabriela Chovancová, Rudolf Hromada

**Affiliations:** a Institute of Parasitology SAS, Hlinkova 3, Košice, 040 01, Slovakia; b I. I. Schmalhausen Institute of Zoology, National Academy of Sciences of Ukraine, B. Khmelnytskogo 15, Kyiv, 01054, Ukraine; c Research Station and Museum of the Tatra National Park, Tatranská Lomnica, 059 60, Slovakia; d University of Veterinary Medicine and Pharmacy in Košice, Komenského 73, Košice, 041 81, Slovakia

**Keywords:** Hymenolepidids, Rodents, Dwarf tapeworm, Rat tapeworm, Europe

## Abstract

*Rodentolepis nana* (syn. *Hymenolepis nana*) and *Hymenolepis diminuta* are hymenolepidid tapeworms of zoonotic importance, but data on their occurrence in free-living rodents in Central Europe are limited. A total of 881 small mammals representing 10 species of the order Rodentia were collected in two areas of Slovakia, the city of Košice and protected areas of the Tatra National Park (TANAP), between 2020 and 2025, and examined for the presence of these tapeworm species. Helminth species were identified using ITS1 and mitochondrial *nad*1 sequence data. *Rodentolepis nana* was detected only in *Rattus norvegicus* from the urban habitat of Košice, with 7 of 97 rats (7.2%) testing positive. *Hymenolepis diminuta* was molecularly confirmed in 10 of 881 examined small mammals (1.1%) and was recorded in *R. norvegicus* (9.3%, 9/97) and *Apodemus agrarius* (1.1%, 1/87). To our knowledge, this study provides the first molecularly confirmed records of both tapeworm species in free-living rodents in Slovakia and extends knowledge on their occurrence in Central Europe.

## Highlights

• Molecular analyses confirmed infections with *Rodentolepis nana* and *Hymenolepis diminuta* in free-living rodents in Slovakia.
• *R. nana* was found only in urban brown rats (*Rattus norvegicus*) from Košice city, with a confirmed occurrence of 10.4%.
• *H. diminuta* had the highest confirmed occurrence in urban brown rats (11.9%) but was also found in *Apodemus agrarius* (1.1%).
• Urban habitats showed the highest occurrence of both tapeworm species, confirming their link to synanthropic rodents.
• Brown rats are important reservoirs and possible sources of human infection with both tapeworms.

## Graphical abstract

**Figure:**
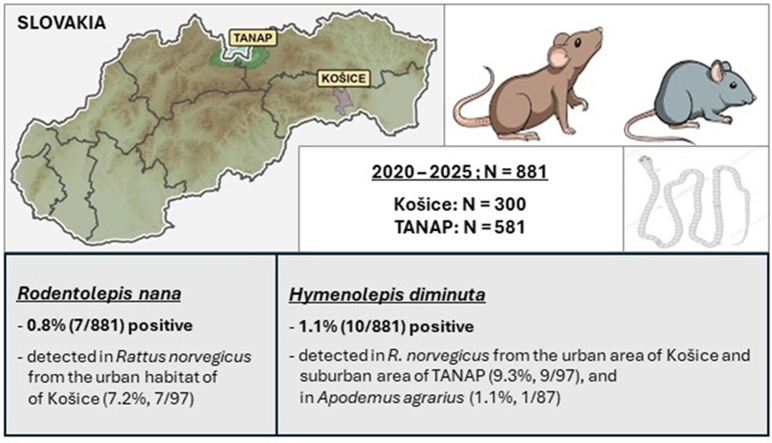

## 1 Introduction

The family Hymenolepididae is a diverse group of cestodes that comprises at least 923 species parasitising mainly birds and mammals (Mariaux et al., 2017). Within this family, *Rodentolepis nana* (syn. *Hymenolepis nana*) and *Hymenolepis diminuta* are of particular interest because of their zoonotic importance.

The dwarf tapeworm (*R. nana*) completes its life cycle in the small intestine of humans, non-human primates, and various rodent species without an intermediate host (direct cycle), although it may also use different arthropods, typically small beetles, as intermediate hosts (indirect cycle) (Thompson, 2015). The eggs are infective immediately after being passed in the stool and can survive for approximately two weeks in the external environment; the parasite has a prepatent period of about four weeks (Acha and Szyfres, 2003). These characteristics facilitate the spread of infection, enabling easy transmission among humans or rodents. Epidemiological evidence suggests that direct human-to-human transmission is the most common route of infection, particularly in areas with poor hygiene and inadequate sanitation (Macnish et al., 2002; Acha and Szyfres, 2003). Nevertheless, infection with *R. nana* is still considered a zoonosis because infected synanthropic or pet rodents, such as mice, hamsters, and rats, serve as a reservoir of infection (Thompson, 2015). Close physical contact between pet rodents and humans further increases the risk of transmission, especially to children, who often do not practice proper hand hygiene after handling animals (Roble et al., 2012).

The rat tapeworm (*H. diminuta*) exhibits only an indirect life cycle. The most common definitive hosts are synanthropic rats, such as *Rattus norvegicus* and *R. rattus*, but the host range also includes humans, several other rodent and insectivore species, primates, and dogs (Burt, 1980). The spectrum of intermediate hosts encompasses more than 60 insect species, primarily representatives of the orders Myriapoda and Insecta (Burt, 1980; Shostak, 2014). Human infection occurs after accidental ingestion of infected insects carrying tapeworm cysticercoids in their body cavities (Bogitsh et al., 2012)*.* This is also why human *H. diminuta* infections are less frequent than those caused by *R. nana*, with most cases recorded in children and in malnourished or immunocompromised individuals (Montgomery and Richards, 2018; Rajeev et al., 2022).

Data on the occurrence of *R. nana* in free-living species in European countries are sporadic, and in the last 15 years, only a few studies on this topic have been published (e.g. Galán-Puchades et al., 2018; Dini et al., 2024; Juhász et al., 2024; Fuentes et al., 2025). A previous preliminary study in Slovakia found one *R. nana*-positive urban brown rat (*R*. *norvegicus*) among 10 urban rodents and 220 different small mammals from the Tatra National Park (Jarošová et al., 2018). Apart from this conference proceeding, *R. nana* has not been recorded in wild or synanthropic rodents in Slovakia since at least 1955 (Syrota et al., 2025).

In contrast, *H. diminuta* has been documented in Slovakia in a wide range of rodent species (*Apodemus agrarius*, *A. flavicollis*, *A. sylvaticus*, *A. uralensis*, *Eliomys quercinus*, *Glis glis*, *Microtus agrestis*, *M. arvalis*, *M. oeconomus*, *M. subterraneus*, *M. tatricus*, *M. musculus*, *M*. *spicilegus*, *Myodes glareolus*, *R*. *norvegicus*, and *Spermophilus citellus*) (Syrota et al., 2025). However, most European studies on *H. diminuta* focus primarily on synanthropic rodents, with rats being the most frequently studied hosts (e.g. Gliga et al., 2020; Dini et al., 2024; Juhász et al., 2024).

Since information on the occurrence of *R. nana* and *H*. *diminuta* in free-living rodents remains limited, the study aimed to document and confirm molecularly their presence in two geographically distinct environments, urban areas and less disturbed habitats within a national park, in Slovakia.

## 2 Materials and methods

### 2.1 Study areas

**Fig. 1:**
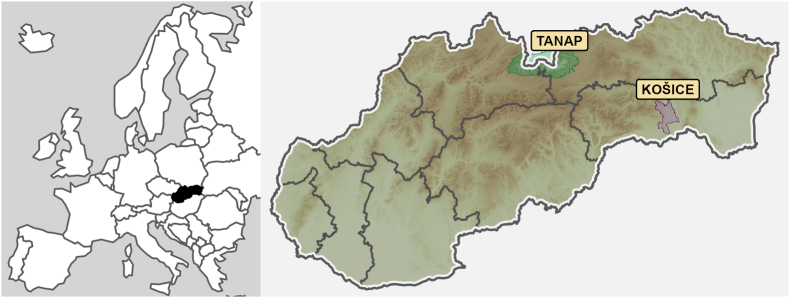
Geographical areas of origin for the sampled rodents in Slovakia: Tatra National Park (TANAP) and the municipality of Košice.

The study on free-living rodents was carried out in two areas of Slovakia with different landscapes and climatic conditions (Fig. 1). The first area comprised rural and suburban areas of the Tatra National Park (TANAP). This is a high-mountain park located in the Carpathian Mountains, forming the natural border between Slovakia and Poland. TANAP spans 738 km^2^ and is home to a diverse range of flora and fauna, including endemic species. The climate is continental, characterised by an alpine climate, with extreme temperature fluctuations, an average annual temperature of 2.5–3.9 °C, and annual precipitation ranging from 599 to 1498 mm (Hlôška et al., 2016).

The second area included suburban and urban localities: the city of Košice and its vicinity. Košice, with approximately 230,000 inhabitants, is the second-largest city in Slovakia and is situated in the eastern part of the country within the Eastern Slovak Basin. The climate is humid continental, with warm summers, cold winters, and a distribution of precipitation throughout the year. The average annual temperature is approximately 9.3 °C, and annual precipitation is about 830–844 mm (En-climate-data, 2025).

For the purpose of the study, forested areas of TANAP located outside towns and villages with low human population density were classified as rural; forests and parks in peripheral parts of Košice and peripheral parts of villages within TANAP were considered suburban, and areas situated directly in the city of Košice were reported as urban.

### 2.2 Collection of small mammals

The rodent specimens analysed in this study were obtained as part of a research project focused on zoonotic pathogens in free-living small mammals in Slovakia. Therefore, portions of the samples used in the present study have previously contributed to studies investigating other parasite groups (Miterpáková et al., 2024; Hurníková et al., 2025; Víchová et al., 2025).

Standard live and snap traps set up in lines with wicks soaked in an oil-and-nut mixture used as bait were used to catch animals. In protected areas of the Slovak part of the TANAP, sampling was performed by employees of the TANAP Museum and Research Station in Tatranská Lomnica. In the municipality of Košice, the sampled animals included individuals captured during pest control operations and those found dead or road-killed. Additionally, rodents were captured in the peripheral (suburban) districts of Košice. The sampling was in line with the permit from the Ministry of Environment of the Slovak Republic No. 498/2018-6.3.

The captured animals were humanely euthanised and delivered to the Institute of Parasitology of the Slovak Academy of Sciences in Košice. Altogether, the examined material comprised 881 rodents collected between 2020 and 2025. The sample included 10 species of the order Rodentia: black-striped field mouse (*Apodemus agrarius*), yellow-necked mouse (*Apodemus flavicollis*), bank vole (*Myodes glareolus*), common vole (*Microtus arvalis*), field vole (*Microtus agrestis*), water vole (*Arvicola amphibius*), harvest mouse (*Micromys minutus*), house mouse (*Mus musculus*), brown rat (*Rattus norvegicus*), and common hamster (*Cricetus cricetus*).

Overall, 581 individual rodents were collected from suburban and rural localities in the TANAP, and 300 animals were trapped in the suburban and urban territories of Košice (Table 1).

**Table 1:** Rodent species (Rodentia) from urban, suburban, and rural localities of Košice and the Tatra National Park (TANAP) between 2020 and 2025.

Species	Košice		TANAP		Total
	Urban	Suburban	Suburban	Rural	
*Apodemus agrarius*	19	32	8	28	87
*Apodemus flavicollis*	17	51	93	225	386
*Arvicola amphibius*	–	–	1	–	1
*Cricetus cricetus*	2	–	–	–	2
*Micromys minutus*	–	–	1	–	1
*Microtus arvalis*	17	1	6	3	27
*Microtus agrestis*	–	–	2	6	8
*Mus musculus*	32	5	59	13	109
*Myodes glareolus*	4	45	18	96	163
*Rattus norvegicus*	67	8	22	–	97
Total	158	142	210	371	881

*Note*: Dashes (−) indicate that no individuals were captured in the respective locality.

### 2.3 Helminthological examination of small mammals

During necropsy, the small and large intestines of the sampled animals were examined under a stereomicroscope. Initial helminthological examination revealed tapeworms, presumptively identified as hymenolepidids, in 56 of the 881 rodents examined. These specimens were recovered from 7 host species: *A. agrarius* (*n = *10), *A. flavicollis* (*n = *7), *M. glareolus* (*n = *10), *M. arvalis* (*n = *1), *M. agrestis* (*n = *3), *M. musculus* (*n = *1), and *R. norvegicus* (*n = *24).

Only presumptive hymenolepidid cestodes were considered further in the present study, with one specimen selected per host for molecular analysis; other helminth taxa were outside the scope of the study.

### 2.4 Molecular analyses

To enable reliable species identification, all 56 specimens were subjected to molecular analysis. Genomic DNA was extracted using the Thermo Scientific GeneJET Genomic DNA Purification Kit (Thermo Fisher Scientific Baltics VAB, Vilnius, Lithuania). PCR reactions were performed using 5× FIREPol® Master Mix Ready to Load (SOLIS Biodyne, Tartu, Estonia). For identification of *R. nana* and *H. diminuta*, PCR-based analyses targeted either a fragment of the internal transcribed spacer 1 (ITS1) or a fragment of the mitochondrial *nad*1 gene (Table 2). The marker selected for sequencing depended on amplification success. Positive PCR products were purified using the ExoSAP IT™ PCR Express Product Cleanup Reagent (Thermo Fisher Scientific, Waltham, MA, USA). Amplicons were sequenced in both directions using the same primers as for PCR amplification. Additional PCR assays, a 1600 bp fragment of the *cox*1 gene (Chambier et al., 2019), a 444 bp fragment of the *cox*1 gene (Okamoto et al., 1997), and a 670 bp fragment of the internal transcribed spacer 2 (ITS2) (Okamoto et al., 1997), were performed as alternative approaches to obtain additional data. However, these assays did not yield readable sequences suitable for further analysis.

**Table 2:** Primers used for amplification of DNA fragments of *Rodentolepis nana* and *Hymenolepis diminuta*.

Target fragment	Primer name	Primer sequence (5′–3′)	T (°C)	Reference
ITS1 (∼650 bp)	F3	GCGGAAGGATCATTACACGTT	63	Macnish et al. (2002)
	R3	GCTCGACTCTTCATCGATCCACG		
*nad*1 (∼850 bp)	Cyclo_nad1F	GGNTATTSTCARTNTCGTAAGGG	55	Littlewood et al. (2008)
	Cyclo_trnNR	TTCYTGAAGTTAACAGCATCA		

*Abbreviation*: T, annealing temperature.

### 2.5 Data processing and analysis

The scientific names of species and higher taxa are used according to the GBIF Backbone Taxonomy (https://www.gbif.org; accessed on 4 April 2026). Geneious Prime v.2026.0.2. was used for trimming and assembling sequencing chromatograms. For species identification, the newly obtained sequences were aligned with reference sequences of *R. nana* and *H. diminuta* from peer-reviewed studies (von Nickisch-Rosenegk et al., 2001; Macnish et al., 2002; Cheng et al., 2016), and pairwise sequence identity was used to assign the sequences to species. All sequences supporting the findings of the present study were submitted to GenBank (PZ201025-PZ201030, PZ201840-PZ201850).

Descriptive statistics were calculated in R v.4.4.2 (R Core Team, 2024), which was also used for map preparation. The occurrence of tapeworms was expressed as the percentage of hosts with molecularly confirmed records among the total number of examined individuals of a certain group. Ninety-five percent confidence intervals (CI) were calculated in the *stats* package v.4.4.2 using the Clopper-Pearson method. The data for preparing the map were obtained *via* the following packages: *giscoR* v.1.1.0, *rnaturalearth* v.1.2.0, and *elevatr* v.0.99.1 (Hollister et al., 2025; Hernangómez, 2026; Massicotte and South, 2026). Spatial data were processed using the *sf* v.1.0.21 and the *terra* v.1.8.70 packages (Pebesma, 2018; Hijmans, 2025). All spatial data were projected to the ETRS89/LAEA Europe coordinate reference system (EPSG:3035). The final map was assembled using *ggplot2* v.4.0.0 and *patchwork* v.1.3.2 packages (Wickham, 2016; Pedersen, 2025).

## 3 Results

### 3.1 Occurrence of *Rodentolepis nana*

Of 56 tapeworm specimens subjected to molecular analysis, seven sequences of *R. nana* were obtained (Table 3). The ITS1 sequences (aligned length 556 bp) showed high similarity (99.16–99.53%) to a published *R. nana* reference sequence (GenBank: AF461124), with the Slovak isolates sharing 99.64–100% identity among themselves. Similarly, the *nad*1 sequences (aligned length 529 bp) exhibited high similarity (99.62%) to the *R. nana* reference sequence (GenBank: KT951722) with an intraspecific identity of 98.49% among the Slovak isolates. Together, these data support taxonomic assignment of the isolates to *R. nana*.

**Table 3:** GenBank accession numbers and metadata for new sequences of *Hymenolepis diminuta* and *Rodentolepis nana* from rodents in Slovakia.

GenBank ID	Host species	Year	Locality	Habitat	Locus
***H. diminuta***					
PZ201840	*R. norvegicus*	2023	TANAP (Spišská Sobota)	Suburban	ITS1
PZ201841	*R. norvegicus*	2024	Košice (Košice city)	Urban	ITS1
PZ201842	*R. norvegicus*	2022	Košice (Košice city)	Urban	ITS1
PZ201843	*R. norvegicus*	2022	Košice (Košice city)	Urban	ITS1
PZ201844	*R. norvegicus*	2021	Košice (Košice city)	Urban	ITS1
PZ201845	*R. norvegicus*	2022	Košice (Košice city)	Urban	ITS1
PZ201025	*R. norvegicus*	2023	Košice (Košice city)	Urban	*nad*1
PZ201026	*A. agrarius*	2022	TANAP (Tatranská Kotlina, Flak)	Rural	*nad*1
PZ201027	*R. norvegicus*	2023	Košice (Košice city)	Urban	*nad*1
PZ201028	*R. norvegicus*	2020	Košice (Košice city)	Urban	*nad*1
***R. nana***					
PZ201846	*R. norvegicus*	2023	Košice (Kavečany)	Urban	ITS1
PZ201847	*R. norvegicus*	2023	Košice (Krásna nad Hornádom)	Urban	ITS1
PZ201848	*R. norvegicus*	2020	Košice (Košice city)	Urban	ITS1
PZ201849	*R. norvegicus*	2023	Košice (Košice city)	Urban	ITS1
PZ201850	*R. norvegicus*	2023	Košice (Košice city)	Urban	ITS1
PZ201029	*R. norvegicus*	2021	Košice (Kavečany)	Urban	*nad*1
PZ201030	*R. norvegicus*	2023	Košice (Kavečany)	Urban	*nad*1

Based on molecular results, *R. nana* was detected in 0.8% of rodent specimens (7/881; 95% CI: 0.32–1.63%). The tapeworm was recorded exclusively in brown rats from the urban areas of Košice. The minimum confirmed occurrence in urban brown rats from Košice was 10.4% (7/67; 95% CI: 4.3–20.3%), while the overall minimum confirmed occurrence in brown rats across all study areas was 7.2% (7/97; 95% CI: 2.9–14.3%). No molecularly confirmed records were detected in either brown rats or any other host species in suburban or rural localities.

### 3.2 Occurrence of *Hymenolepis diminuta*

A total of 10 sequences of *H. diminuta* were obtained (Table 3). The ITS1 sequences (aligned length 308 bp) showed high similarity (99.68–100%) to a published *H. diminuta* reference sequence (GenBank: AF461125), with the Slovak isolates sharing 99.68–100% identity among themselves. Similarly, the *nad*1 sequences (aligned length 329 bp) exhibited high similarity (99.7–100%) to the *H. diminuta* reference sequence (GenBank: NC_002767). No intraspecific differences were observed among the Slovak isolates. Together, these data support the taxonomic assignment of the obtained isolates to *H. diminuta*.

Thus, based on molecular results, *H. diminuta* was detected in 10 of 881 (1.1%; 95% CI: 0.55–2.08%) rodents examined. Similar to *R. nana*, the tapeworm was mainly confirmed in brown rats (9.3%, 9/97; 95% CI: 4.3–16.9%). In particular, eight molecularly confirmed records were obtained in the urban localities of Košice (11.9%, 8/67; 95% CI: 5.3–22.9%), and one record in a brown rat was obtained in a suburban locality of TANAP (4.5%, 1/22; 95% CI: 0.1–22.8%). The tapeworm was also molecularly confirmed in other host species in the rural areas of TANAP, namely in one individual of *A. agrarius* (1.1%, 1/87; 95% CI: 0.03–6.2%).

When comparing habitats across all host species, the minimum molecularly confirmed occurrence of *H. diminuta* was highest in urban localities (5.1%, 8/158; 95% CI: 2.2–9.7%). Corresponding values were lower in less densely populated habitats, reaching only 0.3% (1/352; CI: 0.01–1.57%) in suburban localities and 0.3% (1/371; 95% CI: 0.01–1.49%) in rural localities.

## 4 Discussion

Rodents are highly adaptable and can thrive in a wide range of environments worldwide, making them extremely abundant. They are also known as reservoirs of zoonotic bacteria, viruses, and parasites, which endanger public health by spreading infections through contaminated food or water (Yang et al., 2017).

*Rodentolepis nana* has a cosmopolitan distribution, occurring in both resource-rich and resource-limited settings, and is likely the most common tapeworm causing zoonotic infections globally, especially among children living in poor hygiene and sanitation conditions (Diemert, 2017; Webb and Cabada, 2017).

In the present study, the number of molecularly confirmed *R. nana* records was low; overall, the helminth was confirmed in 0.8% of 881 examined animals. This tapeworm was confirmed only in urban brown rats, and no records were obtained from suburban or rural rats or from any other examined species. However, when analysing only brown rats, 7.2% were positive, and their positivity in the highly populated urban areas of Košice reached 10.4%. Similarly, in a previous preliminary study in Slovakia, *R. nana* was detected in one brown rat among 10 urban rats (10%), and the other 220 examined small mammals remained negative (Jarošová et al., 2018).

Based on the published evidence available, the prevalence of *R. nana* in wild or free-living rodents in European countries generally ranges between 3% and 17%, with most studies focusing on synanthropic rodent populations (mainly rats) (Foronda et al., 2011; Franssen et al., 2016; Galán-Puchades et al., 2018; Dini et al., 2024; Juhász et al., 2024; Fuentes et al., 2025).

The occurrence values observed in the present study are consistent with this pattern and comparable to those reported from China, where *R. nana* was detected in 6.1% of 114 brown rats captured at farms and granaries (Yang et al., 2017). At the same time, considerably higher prevalence values, reaching 19.6% and even 63.7%, have been reported in rats in studies from Iran and India (Brar et al., 2021; Goudarzi et al., 2021).

Information on *R. nana* in rodent hosts other than rats remains limited. The records from Iran and neighbouring regions show its occurrence in *Mus musculus*, gerbils (*Meriones* spp., *Rhombomys* spp., *Tatera indica*), and several other rodent taxa (*Cricetulus* spp. and *Apodemus* spp.) (Zarei et al., 2016; Ranjbar et al., 2017; Moradpour et al., 2018; Goudarzi et al., 2021).

In contrast to free-living animals, *R. nana* has been reported in several pet rodent species (rats, mice, hamsters, chinchillas), with positivity ranging between 13.9% and 41.7% (d’Ovidio et al., 2015; Jarošová et al., 2020; Brustenga et al., 2023). These findings support the presence of an additional transmission link for the parasite in urban environments.

As for parasitism in humans, the dwarf tapeworm is considered the most frequent cestode (Acha and Szyfres, 2003); however, data on its prevalence in human populations over the last 10–15 years are rare. Recent studies suggest that its overall prevalence in human populations is usually low in high-income countries, based on data from Slovakia, where 0.36% of 9350 examined patients and 1.45% of 275 Roma children were positive (Pipiková et al., 2017; Kašlíková et al., 2018), and the western USA, with 1.5% positivity in patients with gastrointestinal symptoms (Church et al., 2010). However, most studies focused on developing countries, where positivity rates range from 1.2% to 32% (Abdel Hamid et al., 2015; Ul Haq et al., 2015; Goudarzi et al., 2021).

*Hymenolepis diminuta*, on the other hand, occurs in humans less frequently than *R. nana*, mainly due to its indirect life cycle, which requires ingestion of infected insects containing tapeworm cysticercoids (Montgomery and Richards, 2018). Worldwide, 1561 cases were reported between 1810 and 2018 (Panti-May et al., 2020), but only 104 laboratory-confirmed cases were reported in 50 European countries (Mijatović et al., 2024). Based on the worldwide overview published by Panti-May et al. (2020), the prevalence of *H. diminuta* in humans was low, ranging from 0.002% to 8.22%.

Epidemiological surveys of *H*. *diminuta* in free-living animals also focused mainly on synanthropic rodents, rats and mice*,* and only limited data are available on its occurrence in European countries. In the present study, the overall proportion of molecularly confirmed records in a mixed sample of small mammals was low (1.3%). However, the values observed in brown rats (9.3%), particularly in urban habitats (11.9%), were broadly comparable to those reported in previous studies from other European countries, where the positivity ranges from 2.27% to over 50.0% (Milazzo et al., 2003; Foronda et al., 2011; Barman et al., 2020; Brar et al., 2021; Dini et al., 2024). Similarly to the present study, higher positivity (50.0%) was reported in rats from farms, while only 10.2% and 10.5% of animals from rural and suburban areas were positive in the Netherlands (Franssen et al., 2016).

The pattern observed in the present study, together with data reported in the literature, supports a close association of *R. nana* and *H. diminuta* with synanthropic rat populations in human-modified environments, where rodents, especially rats (*Rattus* spp.) and mice (*M. musculus*), are well adapted to coexist due to the availability of food resources, such as stored food and garbage. Their omnivorous feeding habits, ability to use human infrastructure, and lack of natural predators enable them to reach high population densities and promote parasite transmission. Moreover, coprophagy, a natural rodent behaviour feature, facilitates the completion of the direct life cycle of *R. nana.* Consequently, high rodent densities, combined with proximity to human settlements or urban environments, significantly increase the risk of *R. nana* and *H. diminuta* infections in rodents and, subsequently, the risk of human infection.

In this study, we chose PCR and subsequent sequencing as the primary and sole approach for species identification due to the condition of the available material. The obtained helminths were not optimal for morphological species-level identification because the rodents had been frozen as whole carcasses before necropsy, and thawing partially damaged the cestode strobilae and scolices. Moreover, a portion of the material originated from animals collected during pest control operations or road-killed individuals and was therefore already partially degraded before freezing. Internal transcribed spacer 1 was used as the first-choice marker because numerous sequences are available in GenBank for comparison. Partial *nad*1 sequences were used when ITS1 chromatograms were unsuitable for reliable interpretation. As an alternative, we also tested assays with primers targeting ITS2 and *cox*1, but the obtained chromatograms were uninterpretable. Taken together, these difficulties indicate that the quality of the material partly limited our study; nevertheless, the species assignments reported here are supported by readable ITS1 and *nad*1 sequences that show high similarity to published data.

It should also be noted that the reported positivity rates may be underestimated, as individual hosts could harbour more than one cestode specimen, but only one specimen per host was analysed molecularly. Therefore, species-specific values should be considered conservative and mixed infections cannot be fully excluded.

## 5 Conclusion

This study provides the first molecularly confirmed occurrence data of two tapeworm species, *R. nana* and *H. diminuta*, in free-living rodents from highly populated and industrialised areas of the city of Košice, and natural localities of the national park (TANAP) in Slovakia. Both tapeworm species were documented mainly in urban brown rats, confirming their relevance in the epidemiology of hymenolepidid tapeworms. These findings are also relevant from a public health perspective, as synanthropic rat populations may be the main source of human infection with *R. nana* and *H. diminuta*, particularly in areas with poor hygiene and sanitation.

## Ethical approval

The captured rodents were humanely euthanised before the carcasses were handled, following authorisation by the Ministry of Environment of the Slovak Republic under permit No. 498/2018-6.3.

## CRediT authorship contribution statement

**Daniela Antolová:** Conceptualization, Validation, Funding, Methodology, Writing - original draft, Writing - review & editing. **Yaroslav Syrota:** Conceptualization, Resources, Methodology, Formal analyses, Writing - review & editing. **Júlia Halapy:**Resources, Methodology. **Michal Stanko:** Resources, Writing - review & editing. **Gabriela Chovancová:** Resources, Writing - review & editing. **Rudolf Hromada:** Resources, Writing - review & editing.

## Statement on the use of AI-assisted technologies

During the preparation of this manuscript, the authors used Grammarly (https://www.grammarly.com/) to correct grammatical errors and improve the text's readability. After using this tool, the authors reviewed and edited the content as needed. The authors take full responsibility for the content of the published article.

## Funding

The research was financially supported by the 10.13039/501100005357Slovak Research and Development Agency under the contract No. APVV-21-0166 and Tubitak-2024-01.

## Declaration of competing interests

The authors declare that they have no known competing financial interests or personal relationships that could have influenced the work reported in this paper.

## Data Availability

All data generated or analysed during this study are included in this published article. The newly generated sequences were submitted to the GenBank database under the accession numbers PZ201840-PZ201850 (ITS1) and PZ201025-PZ201030 (*nad*1).
